# Supplementary material for: Are carbon emissions trading and green financial instruments synergistic? -Comprehensive quantitative research based on content analysis
Source: PLoS One. 2024 Mar 7;19(3):e0298601. doi: 10.1371/journal.pone.0298601 (PMC10919601; doi:10.1371/journal.pone.0298601)
Supplement: S1 Data — (ZIP) [file pone.0298601.s001.zip › Data/地级市Z_F工作报告.zip!/╝¬┴╓/│ñ┤║╩╨/└┤╫╘╣1⁄2╓┌║┼í╛┬φ┐╦╩2╛▌═°í┐_2018.docx]

各位代表：

　　现在，我代表市政府，向大会报告工作，请予审议，并请市政协各位委员提出意见。

　　一、2017年工作回顾

　　2017年是全市上下喜迎党的十九大、经济社会发展取得丰硕成果的一年。一年来，在习近平新时代中国特色社会主义思想指引下，在市委坚强领导下，在市人大、市政协监督支持下，我们紧紧团结和依靠全市人民，抢抓机遇，创新发展，砥砺奋进，圆满完成了经济社会发展主要预期目标，长春老工业基地全面振兴发展取得新的重大进展。

　　——经济运行持续向好。预计地区生产总值增长8%左右，为四年来最好水平。地方级财政收入增长8.3%，快于经济增速。规模以上工业产值突破1万亿元。服务业占GDP比重提高0.6个百分点。

　　——新动能迅速成长。新认定国家级高新技术企业、科技型“小巨人”企业户数分别是上年的2.7倍和3.9倍。大众创业、万众创新蓬勃发展，平均每天新设企业超过100户，为经济发展增添了新生力量。

　　——改革开放深入推进。重要领域和关键环节改革取得突破性进展，供给侧结构性改革成效明显。对外开放迈出新步伐，开放平台和通道建设不断加快。经济社会发展活力明显增强。

　　——城市品质显著提升。伊通河综合治理中段主体工程基本完工，为期两年的旧城改造提升工程全面收尾，地铁1号线投入运营。长春获批国家历史文化名城，全国文明城市、国家卫生城市实现“三连冠”。

　　——各项事业全面进步。教育、科技、文化、卫生、体育等领域取得新进展，环境保护、安全生产、市场监管、社会治安不断加强，社会大局保持和谐稳定。

　　——人民生活不断改善。幸福长春行动计划全面完成。城乡居民增收“暖流计划”惠及893万人次。城镇新增就业11.4万人，超出年初预期目标。长春连续十次获评“最具幸福感城市”。

　　一年来，我们主要做了以下工作：

　　一是加快推动经济转型升级。坚持把扩大有效投资摆在经济工作首位，以项目为载体转方式、调结构、补短板，推动新旧发展动能加快转换。全年实施亿元以上项目1311个、10亿元以上项目313个，分别比上年增加161个和28个。一汽大众Q工厂、北斗科技小镇、华为云计算中心、浪潮大数据中心等一批重大项目顺利推进，航天信息、亚泰医药、光电和智能装备等新型产业园区形成产业集聚，规模以上工业、高技术产业、战略性新兴产业产值分别增长10%、20%和15%左右。新增工业产值超百亿企业2户、规模以上工业企业150户。长春推动实施“中国制造2025”、促进工业稳增长和转型升级工作获国务院通报表扬。服务业增速持续高于一产、二产，经济增长贡献率突破50%。一批商业综合体建成开业，新增商业商务面积近400万平方米。成功举办汽博会、农博会、雕塑大会、电商峰会、创业就业博览会和东北振兴论坛，长春获评“中国十佳品牌会展城市”。龙嘉国际机场年旅客吞吐量突破千万人次。东北首家民营银行亿联银行正式开业，新引进两家域外金融机构。3户企业上市，7户企业新三板挂牌。国家文化消费试点成效显著。“全国绿色有机农业示范市”创建工作扎实推进，现代农业五大实验区建设进展顺利。种植业结构稳中调优，粮食生产再获丰收。品牌农业、高效特色农业、都市休闲农业、健康养殖业初具规模。免疫无口蹄疫区建设通过国家验收。农民合作社数量、土地流转面积持续扩大。农机化率稳步提升。

　　二是坚持以创新引领发展。科技领域一批创新成果达到国际先进水平，长客复兴号新一代高铁上线运营，吉林一号在轨运行卫星达到8颗。全市技术合同交易额突破200亿元，比上年翻一番。科技进步贡献率比上年提高0.9个百分点。互联网与各行业加速融合，新产业、新业态快速成长。各类创新创业基地发展到236个，在孵企业超过1.4万户。长春新区等4家单位获批国家双创示范基地。创新型城市试点建设通过国家验收。国家民营经济发展改革示范城市建设全面启动，民营经济稳步壮大，总量已占全市半壁江山。成立院士专家联合会，成功举办“院士专家长春行”活动。出台集聚人才“20条新政”，启动招才引智“万人计划”。国家级人力资源服务产业园建成运营。国家七项外籍人才出入境政策在长春新区实施。

　　三是大力深化改革开放。全面深化改革，推出一批标志性、关键性改革举措。持续推进“一门式、一张网”综合改革，进一个门办多件事、办事群众与审批人员不见面、行政审批和公共服务事项全程网上运行成为现实，优化了服务环境、方便了办事创业。全面推行“双随机、一公开”，增强事中事后监管的有效性。国企国资、投融资、财税、司法、供销社、医疗卫生、公共资源交易机构整合等改革扎实推进，为经济社会发展增添了新动力。全面扩大开放，主动融入“一带一路”建设。“长满欧”班列货运量快速增长，中欧班列实现首发，长春国际港开通运营。中粮玉米产业园等一批项目落位，新引进央企和国内500强企业5户，实际引进内外资分别增长17%和14%。工业用地出让量、建设用地征收量分别增长42%和62%。积极推动与天津、杭州对口合作，合作园区建设顺利推进。美国、英国等七国大使相继访问我市，城市对外影响力和知名度不断提升。

　　四是建设现代宜居宜业城市。城市设计、城市总体规划、“多规合一”空间规划三项国家试点取得阶段性成果。三环路以内20个商圈、316个老旧住宅区、600多条街路旧貌换新颜。1100多公里地下管网完成改造。地铁1号线投入运营，地铁2号线一期主体工程全线贯通，轻轨北湖线按计划实施。南湖大桥、自由大桥完成翻建。吉林大路东延长线等一批重要路桥建成通车。打通35条断头路、卡脖路。新建20个立体停车场。启动建设6座城区公园，新建26块大宗绿地，植树造林7598公顷。伊通河中段综合治理基本完成。南溪湿地公园建成开放。建成区黑臭水体治理基本完成。新凯河、串湖、东新开河等流域治理有序推进。全域255条河流、6个湖泊全面落实河长制，共设立各级河长2325名。水生态文明城市建设试点任务基本完成。深入实施大气、水、土壤污染防治行动计划，狠抓节能减排。关停搬迁重污染企业12户，基本淘汰黄标车和建成区10吨以下燃煤小锅炉。深入开展“走遍长春”城市精细化管理专项行动。持续推进道路交通秩序整治。“智慧长春”建设取得新进展。

　　五是推动社会事业协调发展。新增义务教育学校8所、普惠性幼儿园18所，打造“温馨村小”182所，创办新优质学校100所。全面推开“蓓蕾计划”，20万名小学生获得课后免费托管服务。长春全域通过义务教育均衡发展国家验收。市医院扩建工程投入使用。51家城市公立医院取消药品加成，我市被确定为公立医院综合改革首批国家级示范城市。加强基层公共文化服务。规划展览馆、雕塑博物馆建成开放，伪满皇宫博物院跻身国家一级博物馆行列，7处国家文物保护单位完成修缮。农安县五台山遗址考古取得重要发现。创排巡演话剧《黄大年》。实施全民健身计划。成功举办首届长春国际马拉松赛。

　　六是注重保障和改善民生。在财政收支压力加大情况下，努力增加民生投入，广大人民群众有了更多获得感。扎实精准推进脱贫攻坚，又有2.7万贫困人口、124个贫困村实现脱贫。加大就业创业支持力度，城镇登记失业率控制在3.5%左右，就业形势保持稳定。开展农民工工资“精准支付”行动。进一步提高最低工资、城乡低保、养老保险、失业保险、重点优抚对象救助标准。城镇居民、农村居民人均可支配收入增速高于全省平均水平。为3.6万名残疾人提供精准康复服务。7家市级医院专科医疗救助中心为农村贫困患者提供免费治疗。新分配公租房4280套，拆除棚户区119万平方米，完成房屋确权登记1008万平方米。出台中低收入家庭公积金贷款购房新政。创建“公交都市”，新增更新公交车辆546台。新建农村公路640公里，改造农村厕所2.9万户，解决18.2万农村居民饮水安全问题。城市千米社区、农村200平方米以上社区比例分别达到75%和80%。

　　七是推进政府建设和治理创新。依法接受人大及其常委会法律监督、工作监督，自觉接受人民政协民主监督。认真听取各民主党派、工商联以及无党派人士的意见建议。办理市人大议案2件、市人大代表建议205件、市政协建议案2件、市政协提案338件。加强安全生产监管，安全生产事故起数、遇难人数持续下降。创建“国家食品安全示范城市”，食品药品监管能力稳步提升。积极开展“电视问政”。加强信访工作。全面提高局长接待日、市长公开电话工作水平。强化社会治安综合治理，依法打击各类违法犯罪活动，有力维护了公共安全。积极促进军民融合发展。民族团结、宗教和谐良好局面进一步巩固。

　　扎实推进“两学一做”学习教育常态化制度化，认真落实中央八项规定精神，坚决纠正“四风”。严格依法行政。加强行政监察和审计监督。驰而不息推进党风廉政建设。

　　各位代表，过去一年长春经济社会发展取得的成绩，是市委正确领导的结果，是人大、政协以及社会各界大力支持的结果，是全市广大干部群众团结奋斗的结果。在此，我代表市政府，向全市广大干部群众致以崇高的敬意！向人大代表、政协委员和社会各界人士，向中省直驻长单位、人民解放军和武警驻长部队以及海内外的朋友们，表示衷心的感谢！

　　肯定成绩的同时，也要清醒看到，我们的工作还存在许多不足，也面临不少困难和挑战。推动经济扩大总量、优化结构、提升质量任务繁重，科技创新引领发展作用尚未充分发挥，推进改革开放、激发市场活力的力度需要进一步加大，城市承载能力、基本公共服务还有不少短板，环境污染治理和生态文明建设任重道远，一些群众关心的热点难点问题还没有得到有效解决，影响经济稳定运行、城市安全的潜在风险和隐患仍然较多。政府职能转变还不到位，软环境建设需要持续发力，廉政建设和作风建设仍需加强。我们一定要直面挑战，敢于担当，全力以赴做好政府工作，不辜负人民的期望和重托。

　　二、2018年重点工作

　　今年是全面贯彻落实党的十九大精神开局之年，是改革开放40周年，是决胜全面建成小康社会、实施“十三五”规划承上启下的关键一年。做好今年工作，要适应新时代、聚焦新目标、落实新部署，在新起点上开启长春振兴发展的新篇章。

　　——必须放眼未来对标高位，牢牢把握事关全局和长远的重大战略部署。把各项任务放到2020年全面建成小康社会、加快长春老工业基地全面振兴发展、建设东北亚区域性中心城市的目标体系中进行定位安排，坚定信心，持续用力，一步一个脚印向前迈进。

　　——必须深入贯彻新发展理念，推动经济转向高质量发展阶段。坚持质量第一、效率优先，以改革创新推动转型升级，努力实现创新成为第一动力、协调成为内生特点、绿色成为普遍形态、开放成为必由之路、共享成为根本目的的发展。

　　——必须把握稳中求进工作总基调，正确处理“稳”和“进”的关系。稳是大局，进是目的。在确保经济运行质量和社会大局稳定的前提下，立足长春发展不平衡不充分的实际，在经济发展、改革开放、城市建设、社会治理等方面加大进的力度，进而有序，进而有为，不断取得新突破。

　　——必须跨越经济发展现阶段关口，打好防范化解重大风险、精准脱贫、污染防治三大攻坚战。坚持靶向聚焦抓重点、多措并举补短板、合力攻坚强弱项，守住防控风险底线，啃下脱贫攻坚“硬骨头”，以更大力度改善生态环境，确保长春发展提质增效、行稳致远。

　　——必须坚持以人民为中心的发展思想，不断促进人的全面发展。把人民对美好生活的向往作为奋斗目标，把增进民生福祉作为发展的根本目的，抓住群众最关心最直接最现实的利益问题，多谋民生之利、多解民生之忧，不断增强群众的获得感、幸福感和安全感。

　　今年政府工作的总体思路是：高举习近平新时代中国特色社会主义思想伟大旗帜，深入贯彻党的十九大精神，认真落实省委十一届二次全会、市委十三届三次全会要求，坚持稳中求进工作总基调，坚持新发展理念，按照高质量发展的要求，统筹推进“五位一体”总体布局和协调推进“四个全面”战略布局，以供给侧结构性改革为主线，统筹推进稳增长、促改革、调结构、惠民生各项工作，大力推进改革开放，推动质量变革、效率变革、动力变革，打好防范化解重大风险、精准脱贫、污染防治攻坚战，促进经济社会持续健康发展，加快建设东北亚区域性中心城市，不断开创新时代长春老工业基地全面振兴发展新局面。

　　全市经济社会发展的主要预期目标是：地区生产总值增长7.8%左右，规模以上工业产值增长8.5%以上，服务业增加值增长8.5%以上，固定资产投资增长10%以上，社会消费品零售总额增长10%以上，地方级财政收入增长7%以上，城乡居民收入与经济增长基本同步，城镇登记失业率控制在4%以内，城镇调查失业率实现稳中有降。这些指标安排，综合考虑了外部环境、现实支撑和区域发展态势，有利于促进就业、引导预期。实际工作中，我们要重视速度，更要重视质量，在质的大幅提升中实现量的有效增长。

　　今年要着重做好七个方面工作：

　　（一）深入实施创新驱动发展战略，推动实体经济优化结构，不断提高质量、效益和竞争力

　　提升科技创新能力。加强政、产、学、研、用、金、介协同创新，促进科技与经济深度融合，争创国家创新型城市。滚动实施10个重大科技攻关、10个重大科技成果转化项目，为100户科技型企业提供精准服务，新认定国家级高新技术企业、科技型“小巨人”企业分别达到200户以上。依托在长大专院校、科研院所，推动校企合作、产研融合。培植壮大北湖科技园等创新园区。发挥科技大市场和科技金融创新中心作用。技术合同交易额增长30%。实施更加有效的人才引进政策，建设国内一流的人力资源服务产业园，为高层次人才、急需紧缺人才及符合条件高校毕业生提供人才公寓。落实科技成果处置权、收益权和股权期权分红等激励政策，激发科研人员积极性、创造性。

　　加快制造业优化升级。以更有力措施促进互联网、大数据、人工智能与制造业深度融合，争创“中国制造2025”国家级示范区。滚动实施10个智能化工厂、20个数字化车间和50条自动化生产线建设，抓好商业遥感卫星、智能机器人、数控设备、半导体照明显示等重大产业化项目，加快推动航天信息、大数据等产业园区建设，支持现有企业实施技术改造，工业投资增长10%左右。全力支持一汽发展自主品牌、新能源、智能网联汽车，鼓励长客、中粮、皓月、大成等企业创新发展，带动三大支柱产业向高端化迈进。加快培育先进装备制造、光电信息、生物及医药健康、新能源汽车、新材料、大数据六大战略性新兴产业，完成投资增长15%以上。

　　推动服务业转型发展。深化“十三五”国家服务业综合改革试点。积极培育共享经济、数字经济、平台经济，加快发展研发设计、检验检测、融资租赁等生产性服务业，做大做强电子商务、服务外包、文化创意、动漫设计等新兴行业。运用新技术、新业态、新模式，改造提升商贸物流、旅游休闲、医疗健康、文化娱乐、会议展览等传统服务业。建设东北亚区域性金融服务中心，搭建“一站式”金融服务平台，支持企业上市、挂牌和再融资。加快建立多主体供应、多渠道保障、租购并举的住房制度。合理增加住宅用地，规范开发、销售、中介等行为，遏制房价过快上涨。积极引导文化等新兴消费。推进国家首批供应链体系试点城市建设，开展“放心消费在长春”活动，增强消费拉动经济增长的基础作用。建设“智慧长春”，推动“互联网+”深入发展，让企业广泛受益、群众普遍受惠。

　　转变农业生产方式。坚持质量兴农、绿色兴农，持续创建“全国绿色有机农业示范市”，推动农业发展由产量导向转为质量效益导向。打造一批优质特色种养基地，建设20个绿色有机农业示范区、30个棚膜经济园区、200个标准化养殖示范区。支持农安创建国家农村产业融合发展示范园。新增无公害、绿色、有机和地理标志农产品70个，提升“长春大米”等品牌知名度。大力发展农产品精深加工业。实施化肥农药减量行动，推广畜牧业无抗养殖技术。加快建设高标准基本农田，推广保护性耕作技术，稳定粮食综合生产能力。培育发展农民合作社、家庭农场等新型经营主体，土地流转面积占比增加4个百分点，农机化率提高2个百分点。

　　全面提升质量水平。实施质量强市战略，广泛开展质量提升行动，评选首届“市长质量奖”，争创全国质量强市示范市。大力弘扬工匠精神，厚植工匠文化，完善激励机制，培育众多长春工匠，打造更多长春品牌，推动长春经济发展进入质量时代。

　　（二）坚持以供给侧结构性改革为主线，激发企业活力和创造力，增强经济发展内生动力

　　推动简政放权、放管结合、优化服务改革向纵深发展。打造“一门式、一张网”2.0版，促进行政审批和公共服务提速增效。深化商事制度改革，推动“证照分离”、“照后减证”、“N证联办”，破解“准入不准营”的难题。优化基本建设项目并联审批，在具备条件的开发区探索开展“零审批”试点。加快政务信息系统整合共享，尽快实现网络、数据、业务互联互通，让群众和企业好办事、少跑腿、不添堵。完善事中事后监管制度，强化“双随机、一公开”，推进综合行政执法。实施企业信用信息统一归集、依法公示、联合惩戒、社会监督，争创国家信用体系建设试点城市。我们一定要让企业和群众更多感受到“放管服”改革成效，除烦苛之弊、施公平之策、开便利之门。

　　激发各类市场主体活力。加快国有经济布局优化和结构调整，完善各类国有资产管理体制，着力提高国有资本运营效率和水平。完成驻长央企“三供一业”分离移交。深入推进国家民营经济发展改革示范，新登记民营企业2万户以上，民营经济主营业务收入增长10%以上。实施百强民营企业、百户“专精特新”中小企业培育工程，规模以上工业、服务业企业分别新增100户、300户以上。建设国家双创示范基地，新建科技孵化器、众创空间、创业孵化基地30个左右，在孵企业增加2000户。

　　加快破除体制机制障碍。深化投融资体制改革，完善政府与社会资本合作模式，激发民间投资活力。发挥金融控股集团作用，运用投资基金吸引社会资本投向重点领域和关键环节。规范政府举债行为，严控增量、消化存量，有效防控债务风险。建立地方金融监管、风险防范处置体系，有效防控金融风险。深化农村金融综合改革试点。推进农村集体产权制度改革，完善农村土地确权颁证工作。推进教育、文化和事业单位改革，深化出租车、公交车行业改革，进一步释放社会领域发展潜力。

　　拓展企业域外发展空间。加快长春国际港建设，完善兴隆综保区功能，稳步提升国际货运班列运营质量。加快建设中德、中俄、中白、中古等国际合作园区。支持装备制造企业建立海外营销和研发中心。鼓励新兴产业开展国际认证、商标注册、销售网络建设，提升出口产品附加值。争创国家跨境电商综合试验区。积极争取设立汽车整车进口口岸。

　　全面提升营商环境质量。继续清理规范涉企收费，落实税收优惠政策，有效控制生产要素成本，切实减轻企业负担。依法保障各种所有制经济组织和公民财产权，激励人们创业创新创富，使企业家安心经营、放心投资。加快构建亲清新型政商关系，发挥广大企业家的积极性、主动性。加强软环境治理，对侵害企业合法权益的行为，必须严肃查处、有错必纠。

　　（三）进一步扩大有效投资，优化空间和产业布局，推动经济持续健康发展

　　充分发挥投资的关键作用。我市产业亟需改造升级，基础设施和民生领域还有许多短板，有效投资仍有很大空间。今年要继续突出项目建设，确保实施亿元以上项目1200个以上。加大招商引资力度，完善落实招商政策，强化签约项目跟踪服务，提高项目履约率和资金到位率，实际利用外来资金增长14%以上。

　　优化区域发展格局。加强分类指导、统筹协调，支持城区、县域和开发区立足定位、发挥优势、各展所长，以增量投资推动发展转型升级。城区要突出发展双创基地、特色街区、现代服务业集聚区和都市型工业、高端制造业园区。县域要以更大力度推进新型工业化，培育有特色的产业集群。长春新区要以创新引领发展，不断提高发展水平和辐射带动能力。国家级开发区要集聚创新创业资源，推动先进制造业、现代服务业、战略性新兴产业不断实现新突破。开展开发区体制机制改革创新试点。整合省级工业集中区、工业园区，推进“一县（市）一开发区”改革。鼓励国家级开发区与县域开发区联动发展。加强与天津、杭州对口合作，建设津长产业合作园、津长双创示范基地和吉浙净月服务业发展示范区。

　　全面实施乡村振兴战略。顺应城乡融合发展趋势，支持资本、技术、人才等要素更多向乡村流动。按照中等城市标准抓好县城建设，培育一批特色产业小镇，容纳更多农民工就近就业创业。加快改善农村交通、通信、电网、环保等基础设施。基本完成农村广电网络光纤入户升级改造。新建改造农村公路300公里。坚持不懈搞好“厕所革命”。解决4.5万农村居民饮水安全问题。实施农村人居环境三年行动计划，建设既有现代文明、又具田园风光的美丽乡村。

　　（四）坚持以人民为中心的城市观，以强烈的文化意识指导城市规划、建设和管理，努力打造现代宜居宜业城市

　　突出规划引领，高质量编制新一轮城市总体规划，推进空间规划、城市设计试点。完善“两横三纵”快速路，启动抚长高速人民大街出口改移工程，推进吉林大路快速路、景阳大路三四环连接线、硅谷大街和腾飞大路绕城立交及延长线等路桥建设。再打通一批断头路、卡脖路，建设一批人行过街天桥、立体停车场。加快西客站周边基础设施建设。畅通净月、莲花山与主城区路网联系。建设长春经济圈环线。龙嘉国际机场二期扩建工程竣工。地铁2号线、轻轨北湖线一期建成通车。长春站综合换乘中心投入使用。调整一批公交线路，更新公交车辆400台。加快港湾式停靠站、公交首末站建设。启动建设第六净水厂，继续改造二次供水设施。续建地下综合管廊。新增供热面积500万平方米。新建改造地下管网500公里。发展装配式建筑。新建续建公园7座，新增绿地100公顷，植树造林4000公顷。与一汽合作建设红旗小镇，打造汽车文化历史街区。综合整治城区铁路沿线、城市出入口等重点区域。巩固提升旧城改造成果，建立长效管理机制。持续开展“走遍长春”专项行动，及时修补“城市伤痕”，提高精细化管理水平。强化交通秩序整治，提高城市畅通水平。

　　（五）打好污染防治攻坚战，加大生态环境保护治理力度，推动绿色发展取得新突破

　　良好生态环境是民生所系、民之所盼。我们要制定和实施打好污染防治攻坚战方案，加大投入，强力推进，严格控制污染源头，狠抓末端排放治理，切实整改突出问题，让人民群众真正感受到环境改善的成果。打赢蓝天保卫战，大力调整能源结构，有序推进“煤改气”、“煤改电”，发展冬季清洁取暖。实施20万千瓦以上燃煤发电机组超低排放改造，淘汰20吨以下燃煤锅炉1000台。加大油品升级和机动车尾气治理力度。推广应用新能源汽车。突出抓好秸秆禁烧和综合利用。加大石头口门、新立城两大水源地综合治理力度。新建、续建、提标改造10座污水处理厂。完善黑臭水体治理工程，落实水污染防治责任，努力实现建成区水体长治久清。加快推进伊通河南南段、北北段和串湖、新凯河、东新开河、饮马河等重点流域治理。全面落实河长制，形成巡河、治河、护河常态机制。推动生活垃圾分类投放，启动建设生活垃圾焚烧发电厂，餐厨垃圾处理厂投入使用。开展土地污染详查，严格建设用地准入，加强重金属污染防治。实施退耕还林还草还湿、矿山环境修复。严格环境执法，对环境违法犯罪，必须依法惩治。

　　（六）实施幸福长春行动计划，全力保障改善民生，促进人的全面发展、全体人民共同富裕

　　打好精准脱贫攻坚战。今年再减少农村贫困人口3700人。深入推进产业、劳务、教育扶贫，增强贫困群众自我发展能力。强化对老年人、残疾人、重病患者等特殊贫困人口精准扶贫，因户因人落实救助供养、大病救治、生活兜底保障等措施。已脱贫人口，攻坚期内“脱贫不脱政策”。新产生的贫困人口和返贫人口，要及时纳入帮扶。落实脱贫攻坚责任制，实施最严格的评估考核，严肃查处假脱贫、“被脱贫”、数字脱贫，确保脱贫工作得到群众认可、经得起历史检验。

　　着力扩大就业创业。实施更加积极的就业政策，城镇新增就业11万人。鼓励引导高校毕业生创新创业，切实做好退役军人安置和就业创业服务。加大就业援助力度，扶持城镇困难人员、残疾人就业，确保零就业家庭至少有一人稳定就业。滚动实施“暖流计划”，促进城乡居民持续稳定增收。开展农民工工资“精准支付”行动，确保按时足额发放。

　　强化基本公共服务。办好学前教育、特殊教育、民族教育、继续教育。新建6所义务教育学校、12所普惠性幼儿园。推进“大学区”改革，深化与吉大、东北师大基础教育合作，城区、农村优质学校覆盖面分别达到95%和65%。200所农村学校完成火炉取暖替代改造。着力降低中小学生过重课业负担。强化师德师风建设。加强校园周边环境治理。提高家庭医生签约服务覆盖率。市妇产医院完成异地搬迁，儿童医院新建项目投入使用。深入开展文化惠民活动。博物馆、美术馆、方志馆对外开放，启动少年宫新建、评剧院还建、市图书馆少儿部和伪满皇宫东北沦陷史陈列馆改扩建。建设拖拉机厂文化科创园区。办好电影节、汽博会、农博会、冰雪节等节庆会展活动。提高文物和非物质文化遗产保护利用水平，强化历史文化传承。广泛开展全民健身，大力发展体育事业，高质量办好第二届长春国际马拉松赛和第十八届省运动会。深化群众性精神文明创建活动，不断提高市民素质和社会文明程度。

　　织密织牢社会保障安全网。落实全民参保计划，推动社保扩面征缴。对养老保险缴费困难群体给予助保支持。进一步提高失业金、退休人员养老金、城乡居民基本养老金标准。加大公积金改革力度，降低进城务工人员、新就业大学生购房成本和门槛。加大公租房保障力度。改造2500户夹馅棚户区、7500户连片棚户区。完成农村危房改造。抓好居家和社区养老服务改革试点，启动建设3个托老中心、20个居家养老服务中心，新增养老床位3000张。为1.5万名困难老人购买居家养老服务。扩大医保救助范围，上调低保对象基本医疗住院救助比例。建立农村贫困人口大病医疗多重保障机制。有需求残疾儿童、持证残疾人接受基本康复服务比例达到70%以上。老年人、残疾人优惠乘车卡使用范围扩至地铁、轻轨和郊线公交。

　　（七）加强和创新社会治理，维护社会和谐稳定，实现城市长治久安

　　生命高于一切，安全重于泰山。牢固树立安全发展理念，深入推进安全生产领域改革，完善安全生产责任制，持续创建“国家安全发展示范城市”。完善立体化社会治安防控体系，实施县、乡、村三级公共安全视频监控“雪亮工程”。依法严厉打击各类违法犯罪，保护人民群众人身权、财产权、人格权。持续创建“国家食品安全示范城市”，把好饮食用药安全关口。社会综合治理信息平台投入使用。健全突发事件应急机制，增强防灾减灾能力。

　　推动社会治理创新。健全基层群众自治制度，加强城乡社区治理。城市千米社区、农村200平方米以上社区比例分别达到85%和 90%。充分利用信访机构接待、局长接待日、市长公开电话、读报读网等机制，依法及时就地解决群众合理诉求。落实“七五”普法规划。完善公共法律服务体系，扩大法律援助范围。进一步做好国家安全和民族宗教工作。配合部队完成停止有偿服务工作。全力支持军队和国防建设，深入开展双拥共建，推动军民融合深度发展。

　　各位代表！今年的任务艰巨而繁重，肩负的使命光荣而重大。

　　我们要坚定政治方向，牢固树立“四个意识”，坚决维护以习近平同志为核心的党中央权威，自觉在思想上政治上行动上同党中央保持高度一致。

　　我们要依法全面履职，依法接受人大及其常委会的法律监督、工作监督，自觉接受人民政协的民主监督，严格依法行政，坚持从严治政，努力提高政府法治化水平。

　　我们要转变工作作风，坚决落实中央八项规定精神，持之以恒纠正“四风”，以奋发有为、昂扬向上的精神状态狠抓落实、成就事业。

　　我们要密切联系群众，始终把人民利益摆在第一位，坚持不懈地为群众办实事、解难题、谋福祉，让振兴发展成果更多更公平地惠及广大人民群众。

　　我们要加强队伍建设，深入推进“两学一做”学习教育常态化制度化，开展“不忘初心、牢记使命”主题教育，强化反腐倡廉，规范权力运行，努力建设让人民满意放心的政府。

　　各位代表！新时代要有新气象新作为。让我们更加紧密地团结在以习近平同志为核心的党中央周围，在省委、省政府和市委的坚强领导下，锐意进取、埋头苦干，为推动长春老工业基地全面振兴发展、加快建设东北亚区域性中心城市而努力奋斗！
